# Modeling Users' Activity on Twitter Networks: Validation of Dunbar's Number

**DOI:** 10.1371/journal.pone.0022656

**Published:** 2011-08-03

**Authors:** Bruno Gonçalves, Nicola Perra, Alessandro Vespignani

**Affiliations:** 1 School of Informatics and Computing, Center for Complex Networks and Systems Research, Indiana University, Bloomington, Indiana, United States of America; 2 Pervasive Technology Institute, Indiana University, Bloomington, Indiana, United States of America; 3 Complex Systems Computational Lab, Linkalab, Cagliari, Italy; 4 Institute for Scientific Interchange, Turin, Italy; University of Maribor, Slovenia

## Abstract

Microblogging and mobile devices appear to augment human social capabilities, which raises the question whether they remove cognitive or biological constraints on human communication. In this paper we analyze a dataset of Twitter conversations collected across six months involving 1.7 million individuals and test the theoretical cognitive limit on the number of stable social relationships known as Dunbar's number. We find that the data are in agreement with Dunbar's result; users can entertain a maximum of 100–200 stable relationships. Thus, the ‘economy of attention’ is limited in the online world by cognitive and biological constraints as predicted by Dunbar's theory. We propose a simple model for users' behavior that includes finite priority queuing and time resources that reproduces the observed social behavior.

## Introduction

Recently, the divide between the physical world and online social realities has been blurred by the new possibilities afforded by real-time communication and broadcasting, which appear to greatly enhance our social and cognitive capabilities in establishing and maintaining social relations. The combination of mobile devices with new tools like Twitter, Foursquare, Blippy, Tumblr, Yahoo! Meme, Google Hotspot, etc., are defining a new era in which we can be continuously connected with an ever-increasing number of individuals through constant digital communication composed of small messages and bits of information. Thus, while new data and computational approaches to social science [1], [2], [3] finally enable us to answer a large number of long-standing questions [4], [5], [6], we are also increasingly confronted with new questions related to the way social interaction and communication change in online social environments: What is the impact that modern technology has on social interaction? How do we manage the ever-increasing amount of information that demands our attention?

In 1992, R. I. M. Dunbar [7] measured the correlation between neocortical volume and typical social group size in a wide range of primates and human communities. The result was as surprising as it was far-reaching. The limit imposed by neocortical processing capacity appears to define the number of individuals with whom it is possible to maintain stable interpersonal relationships. Therefore, the size of the brain's neocortex represents a biological constraint on social interaction that limits humans' social network size to between 100 and 200 individuals [8], i.e. Dunbar's number. McCarty et al. [9] independently attempted to measure typical group size using two different methods and obtained a number of 291, roughly twice Dunbar's estimate.

Biological constraints on social interaction go along with other real-world physical limitations. After all, a person's time is finite and each person must make her own choices about how best to use it given the priority of personal preferences, interests, needs, etc. The idea that attention and time are scarce resources led H. Simon [10] to apply standard economic tools to study these constraints and introduce the concept of an Attention Economy with mechanisms similar to our everyday monetary economy. The increasingly fast pace of modern life and overwhelming availability of information has brought a renewed interest in the study of the economy of attention with important applications both in business [11] and the study collective human behavior [12]. On one hand, it can be argued that microblogging tools facilitate the way we handle social interactions and that this results in an online world where human social limits are finally lifted, making predictions such as the Dunbar's number obsolete. Microblogging and online tools, on the other hand, might be analogous to a pocket calculator that, while speeding up the way we can do simple math, does not improve our cognitive capabilities for mathematics. In this case, the basic cognitive limits to social interactions are not surpassed in the digital world. In this paper we show that the latter hypothesis is supported by the analysis of real world data that identify the presence of Dunbar's limit in Twitter, one of the most successful online microblogging tools.

## Materials and Methods

**Figure 1:**
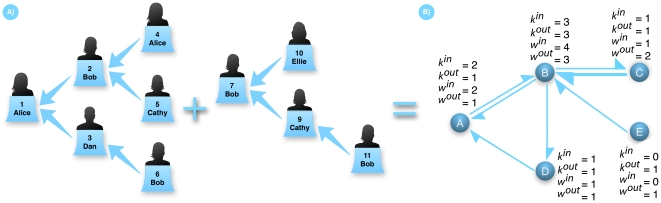
Reply trees and user network. A) The set of all trees is a forest. Each time a user replies, the corresponding tweet is connected to another one, resulting in a tree structure. B) Combining all the trees in the forest and projecting them onto the users results in a directed and weighted network that can be used as a proxy for relationships between users. The number of outgoing (incoming) connections of a given user is called the out (in) degree and is represented by k^out^ (k^in^). The number of messages flowing along each edge is called the degree, w. The probability density function *P(k^out^)* (*P(k^int^)*) indicates the probability that any given node has *k^out^* (*k^in^*) out (in) degree and it is called the out (in) degree distribution and is a measure of node diversity on the network.

Here we analyze a massive dataset of Twitter conversations accrued over the span of six months and investigate the possibility of deviation from Dunbar's number in the number of stable social relations mediated by this tool. The pervasive nature of Twitter, along with its widespread adoption by all layers of society, makes it an ideal proxy for the study of social interactions [13], [14], [15], [16]. We have analyzed over 380 million tweets from which we were able to extract 25 million conversations. Each Twitter conversation takes on the form of a tree of tweets, where each tweet comes as a reply to another. By projecting this forest of trees onto the users that author each tweet, we are able to generate a weighted social network connecting over 1.7 million individuals (see Figure 1).

In the generated network each node corresponds to a single user. The out-degree of the nodes is the number of users the node replies to, while the in-degree corresponds to the number of different nodes it receives a reply from. When A follows B, A subscribes to receive all the updates published by B. A is then one of B's followers and B is one of A's friends. Previous studies have mostly focused on the network induced by this follower-friend relationship [15], [17], [18], [19]. In any study about stable social relations in online media, as indicated by studies about Dunbar's number, it is important to discount occasional social interactions. For this reason we focus on stronger relationships [13] in our study, considering just active communication from one user to another by means of a genuine social interaction between them. In our network [20], [21] we introduce the weight *w_ij_* of each edge, defined as the number of times user *i* replies to user *j* as a direct measurement of the interaction strength between two users and stable relations will be those with a large weight. A simple way to measure this effect is to calculate the average weight of each interaction by a user as a function of his total number of interactions. Users that have only recently joined Twitter will have few friends and very few interactions with them. As time goes by, stable users will acquire more and more friends, but the number of replies that they send to other users will increase consistently only in stable social interactions. Eventually, a point is reached where the number of contacts surpasses the user's ability to keep in contact with them.

**Figure 2:**
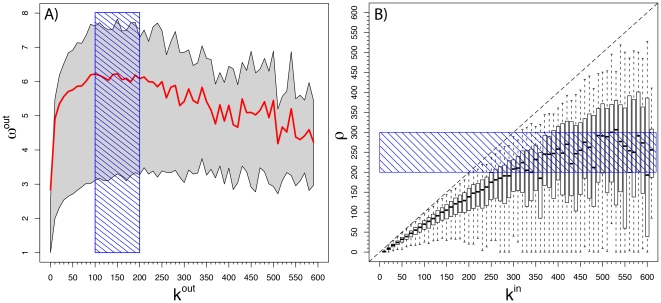
Connection weight and Reciprocated connections. A) Out-weight as a function of the out-degree. The average weight of each outward connection gradually increases until it reaches a maximum near 150–200 contacts, signaling that a maximum level of social activity has been reached. Above this point, an increase in the number of contacts can no longer be sustained with the same amount of dedication to each. The red line corresponds to the average out-weight, while the gray shaded area illustrates the 50% confidence interval. B) Number of reciprocated connections, ρ, as a function of *k^in^.* As the number of people demanding our attention increases, it will eventually saturate our ability to reply leading to the flat behavior displayed in the dashed region.

This saturation process will necessarily lead to some relationships being more valued than others. Each individual tries to optimize her resources by prioritizing these interactions. To quantify the strength of these interactions, we studied the quantity *ω_i_^out^* , defined as the average social strength of active initiate relationship:
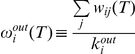
This quantity corresponds to the average weight per outgoing edge of each individual where T represents the time window for data aggregation. We measure this quantity in our data set as shown in Figure 2A. The data shows that this quantity reaches a maximum between 100 and 200 friends, in agreement with Dunbar's prediction (see figure 2A). This finding suggests that even though modern social networks help us to log all the people with whom we meet and interact, they are unable to overcome the biological and physical constraints that limit stable social relations. In Figure 2B, we plot 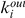, the number of reciprocated connections, as a function of the number of the in-degree. 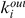
saturates between 200 and 300 even though the number of incoming connections continues to increase. This saturation indicates that after this point the system is in a new regime; new connections can be reciprocated, but at a much smaller rate than before. This can be accounted for by spurious exchanges we make with some contacts with whom we do not maintain an active relationship.

If we assume that biological and time constraints are the key ingredients in fixing Dunbar's number, then it is interesting to define a minimal agent model entailing those key features in the form of a dynamical process. We consider a static network where each agent (node) *i* is connected to its nearest neighbors *j* through two directed edges. Whenever a message is sent from node *i* to node *j*, the weight of the (*i, j*) edge, *w_ij_* is increased by one. The total activity of each user is given by the sum over all of its outgoing edges and the out-degree is equal to the in-degree edge. In this way we are able to distinguish between incoming and outgoing messages. Indeed, in Twitter user relationships are directed and not always reciprocal. One of Twitter's features is to always show replies, even from users we do not explicitly follow. In this way, conversations can flow back and forth between users regardless of whether or not they have an explicit mutual follower relationship.

Agents communicate with each other by replying to messages. When agent *i* receives a message the message is placed in an internal queue that allows up to *q_max_* messages to be handled at each time step. In the presence of finite resources each agent has to make informed decisions about which are the most important messages to answer. This is a direct consequence of the physical constraints that we model by assuming messages are stored according to a priority set proportional to the total degree of the sender *j.* In this way, we implicitly assume that the degree is a proxy for popular and socially active agents who are more likely to be answered. A user's queue provides a minimal and simplistic representation of the finite cognitive and time capabilities that each user has by imposing limits and prioritization to active communications. At each time step, each agent goes through its queue and performs the following simple operations:

- The agent replies to a random number *S_t_* of messages between 0 and the number of messages *q_i_* present in the queue. The messages to be replied to are selected proportionally to the priority of the sending agent (its total degree). A message is then sent to j, the node we are replying to, and the corresponding weight *w_ij_* is incremented by one.
- Messages the agent has replied to are deleted from the queue and all incoming messages are added to the queue in a prioritized order until the number of messages reaches *q_max_*. Messages in excess of *q_max_* are discarded.

The dynamic process is then repeated for a total number of time steps T. In order to initialize the process and take into account the effect of endogenous random effects, each agent can broadcast a message to all of its contacts with some small probability *p*. One may think of this message as a common status change, or a TV appearance, news story, or any other information not necessarily authored by the sending agent. Since these messages are not specifically directed from one user to another, they do not contribute to the weight of the edges through which they flow. We have studied this simple model by using an underlying network of N = 10^5^ nodes and different scale-free topologies. For each simulation T = 2×10^4^ time steps have been considered and the plots are made evaluating the medians among at least 1000 runs.

**Figure 3:**
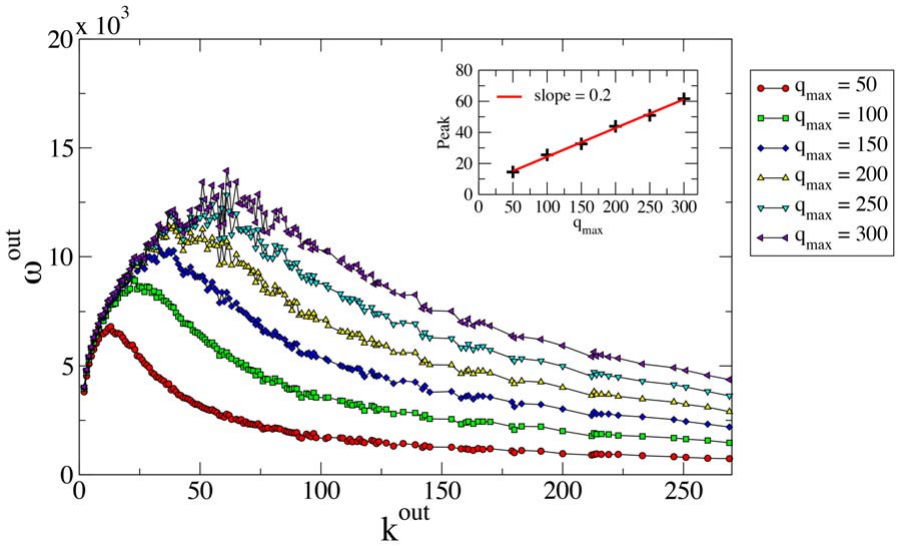
Result of running our model on a heterogeneous network made of *N* = 10^5^, nodes with degree distribution 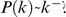 with γ = -2.4 and σ = 10. Different curves correspond to different queue size. The inset shows the linear dependence of the peak on the queue size *q*. Each curve is the median of 1,000 to 2,000 runs of *T = 2×10^4^* time steps. In the inset, we plot the position of the peak as a function of the queue size. The linear relation is clear.

In Figure 3 we report the results of simulations in a directed heavy-tailed network with a power-law tail similar to those observed for the measured network (see Information S1). The figures clearly show a behavior compatible with the empirical data. The peak that maximizes the information output per connection is linearly proportional to *q_max_*_,_ supporting the idea that the physical constraints entailed in the queue's maximum capacity along with the prioritization that gives importance to popular senders are at the origin of the observed behavior. We have also performed an extensive sensitivity analysis on the broadcasting probability p, the time scale *T*, and have investigated the effect of agent heterogeneity by studying populations in where each agent's capacity *q_max,i_* is randomly distributed according to a Gaussian distribution centered around *q_max_* with standard deviation *σ*. In Information S1 we present an extensive discussion concerning the weak effects that variations in the broadcasting probability, p, the time scale, T, and agent heterogeneity have on the obtained results.

## Results and Discussion

**Figure 4:**
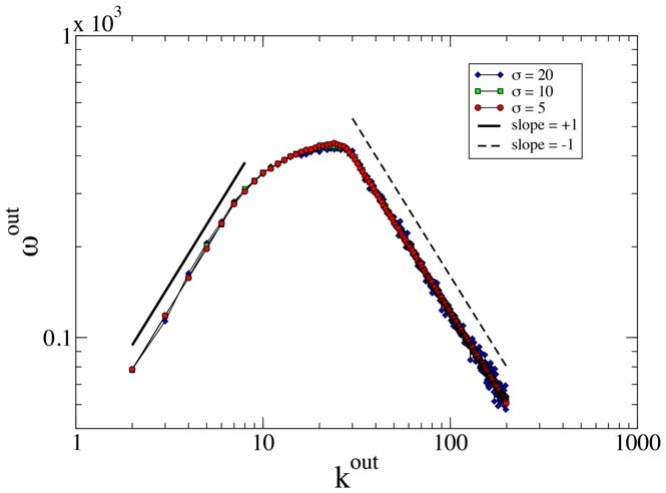
Results for the single user and different values of σ, the inter-user queue size variance. We fixed the average queue size at *q_max,i_ = *50 and extracted the priorities of user neighbors from a power-law statistical distribution with exponent γ = −2.1. For each *k_i_* we run *T = *500 time steps and present the medians among 10^3^ runs.

In order to provide insight into the mechanisms behind the behavior we observe in the model, we consider the point of view of a single user. A set of *k_i_* directed links is assigned to the user *i.* This user can interact with *k_i_/2* other users and their contacts, sending messages to them through *k_i_^out^ = k_i_/2* outgoing links or receiving messages from them through *k_i_^in^ = k_i_/2* incoming links. The dynamics of our model are then applied for *T* time steps. The quantity *ω_i_^out^(T)* is evaluated for different values of *k_i_* (see Figure 4). In this mean-field approach we ignore the dynamics of all users connected to *i*. Instead, we use them as the source of messages that focus our attention to the behavior of a single individual connected to them. The average number of messages that *i* receives at each time step is *<R>∼k_i_^2^*. This is given by the fact that the number of messages each user receives is proportional to the number of connections times the probability that a connected individual sends a message to the agent. The latter probability increases with the popularity of the agent *i* and is proportional to its degree *k_i_*. Two different regimes are therefore found to be a function of *k_i_*.

Given a small number of contacts *k_i_*, the number of messages that the user *i* receives is small with respect to the queue size *q_max_*. At each time step *i* can in principle reply to all received messages. The number of outgoing replies in this regime scales with the number of received messages. For large enough *T* the user will be able to reply to all the received messages *<R>* and the average number of replies for connections will scale as 
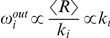
. For a number of contacts larger than the queue size *q_max_*, the user will be unable to reply to all messages in the queue. Once the saturation effect takes place, the user will on average reply to the same number of messages at each time step. The average number of replies per connection will therefore scale as 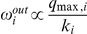. These are indeed the two clear regimes observed in the empirical data and in the model simulations. Furthermore, in Figure 4 we consider the inclusion of Gaussian noise with varying standard deviation *σ* in the queue size *q_max_* of agents. The plots show that different noise levels do not affect the model's behavior. Despite its simplicity, this mean-field analysis clearly shows the key mechanism and ingredients of our model: limitation of resources and prioritization of tasks.

The simple model that we have introduced offers a basic explanation of a seemingly complex phenomena observed in the empirical patterns on Twitter data and offers support to Dunbar's hypothesis of a biological limit to the number of relationships than can be simultaneously maintained by a single individual. The social interaction mechanism we propose: limited attention and internal prioritization of interactions, is sufficiently parsimonious and robust to be applicable to a wide range of social scenarios.

## Supporting Information

Information S1 **Detailed description of the Twitter data, sensitivity analysis of the parameter's model and analytical description of the single user model.**
(PDF)
