# Supplementary material for: Modeling Users' Activity on Twitter Networks: Validation of Dunbar's Number
Source: PLoS One. 2011 Aug 3;6(8):e22656. doi: 10.1371/journal.pone.0022656 (PMC3149601; doi:10.1371/journal.pone.0022656)
Supplement: Information S1 — Detailed description of the Twitter data, sensitivity analysis of the parameter's model and analytical description of the single user model. (PDF) [file pone.0022656.s001.pdf]

Supplementary Information
Modeling users' activity on Twitter networks: validation of Dunbar's number

Bruno Gonçalves, Nicola Perra, Alessandro Vespignani

May 26, 2011

Contents

1 Data Details **1**
1.1 Tree Identification and Projection 1
1.2 Online Conversations 2

2 The Model **2**
2.1 Effect of the Time Window T 4
2.2 Effect of Broadcast Probability p 5
2.3 Effect of Network's Properties 6
2.4 Single User Analytics 8

1 Data Details

Having been granted temporary access to Twitters firehose we mined the stream for over 6 months to identify a large sample of active user accounts. Using the API, we then queried for the complete history of 3 million users, resulting in a total of over 380 million individual tweets covering almost 4 years of user activity on Twitter. Tab. S 1 provides some basic statistics about our dataset.

1.1 Tree Identification and Projection

All tweets in our dataset that constituted a reply were collected. Each such tweet contains information not only about the id of the original tweet but also the user that sent it. Using this information, each reply tweet maps directly to a directed edge. Individual trees can be identified by using depth first search [1] to identify connected components in the resulting tweet-tweet graph. To ensure that the full tree is found and not just a part of it, we treat each link as undirected for the purposes of this identification. In this way we are able to extract the complete tree even if we happen to start on one of the leaves. For each tree the root is then found by locating the node with $k_{in} \equiv 0$, and

Tweets	381,652,990
Timelined Users	3,006,180
Scraping Period	Nov. 20, 2008 – May 29, 2009
Time span	4 years
Trees	25,273,871
Tweets in Trees	81,728,252
User in Trees	1,720,320
User-User Edges	68,459,592

Tab. S 1: Dataset Statistics.

distances from the root are measured by rerunning the DFS algorithm starting from the root and respecting the direction of each edge.

The underlying reply network can be extracted by projecting the tweet trees to a user graph: User A is connected to user B by a directed outgoing edge if A replied to a tweet sent by B. Over time, any pair of users can exchange multiple replies either in a single "conversation" (tree) or through multiple conversations. The number of messages sent from one user to another is used as the weight of the corresponding directed edge and is taken to signify the strength of the connection between the two users, with higher weights representing stronger connections.

1.2 Online Conversations

Each reply creates a connection between two tweets and their authors, so we can define a conversation as a branching process of consecutive replies, resulting in a tree of tweets. From our dataset we extracted and analyzed a forest of over 25 million trees. Trees vary broadly in size and shape, with most conversations remaining small while a few grow to include thousands of tweets and hundreds of users, as shown in Fig. S 1.

A directed user-user network can be built by projecting conversation trees to detail how users interact and establish relationships among themselves. Bidirectional edges signify mutual interactions, with stronger weights implying a more frequent or prolonged interaction between two individuals.

All of our analysis will be performed on this user-user conversation network. We consider a user to have out degree k_{out} if he or she replies to k_{out} other users, regardless of the number of explicit followers or friends the given user has. By focusing on direct interactions we are able to eliminate the confounding effect of users that have tens or hundreds of thousands of followers with whom they have no contact and are able to focus on real person to person interactions [2].

2 The Model

The model that we propose is based on the assumption that the biological and time constraints are the key ingredients in fixing the Dunbar's number. We consider a static network $\mathcal{G}$, characterized by a degree distribution $P(k)$, where each agent (node) i is connected to its nearest neighbors j

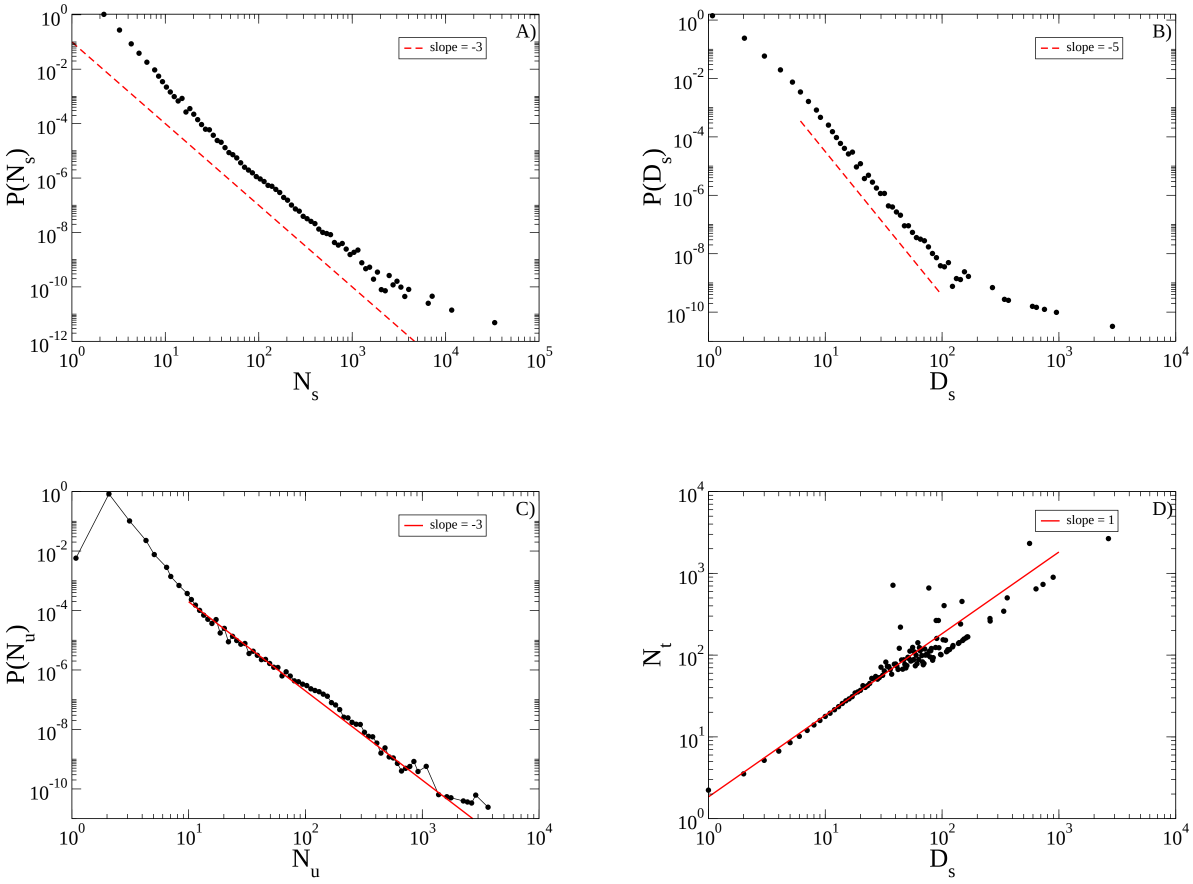

Fig. S 1: Tree characterization. A)Distribution of the number of tweets in a tree. B) Distribution of the number of shells. C) Distribution of the number of users. D) Tree size vs depth. The broad tailed nature of all of these quantities indicates the diversity of behaviors displayed by the users in our dataset.

through two directed edges, $i \rightarrow j$ and $j \rightarrow i$. Whenever a message is sent from node i to node j, the weight of the (i,j) edge, w_{ij} is increased by one. The total activity of each user is given by the sum over all of its outgoing edges and the out degree is identically equal to the in degree:

$$k_i^{out} = k_i^{in} = \frac{k_i}{2}, \ \ \forall i \in \mathcal{G}. \tag{1}$$

We use this framework in order to distinguish between incoming and outgoing messages. Users communicate with each other by replying to messages. When agent i receives a message it places it in an internal queue that allows up to $q_{max,i}$ messages to be handled at each time step. In the presence of finite resources each agent has to make informed decision on what are the most important messages to answer. This is a direct consequence of the physical constraints that we model by assuming that messages are stored according to a priority set proportional to the total degree of the sender j. For each user the basic quantity that we studied is the average number of interactions per connection:

$$\omega_i^{out}(T) = \frac{\sum_j w_{ij}(T)}{k_i^{out}(T)}. \tag{2}$$

At each time step each agent goes through its queue and performs the following simple operations:

1. The agent replies to a random number r of messages between 0 and the number of messages q_i currently present in the queue. The messages to be replied are selected proportionally to the priority of the sending agent, its degree. A message is then sent to j, the node we are replying to, and the corresponding weight w_{ij} is incremented by one.

2. Replied messages are deleted from the queue and all incoming messages are added to the queue in a prioritized order until the number of messages reaches $q_{max,i}$. Messages in excess of q_{max} are discarded. The dynamical process is then repeated for a total number of time steps T. In order to initialize the process and keep into account the effect of endogenous random effects each agent can broadcast a message to all of its contacts with some small probability p. One may think of this message as a common status change, or a TV appearance, news story, or any other information not necessarily authored by the sending agent. Since these messages are not specifically directed from one user to another, they do not contribute to the weight of the edges through which they flow.

It is well known [3] that the following, flowers networks on twitter are well approximated by a power-law degree distributions. For this reason we used as baseline a scale-free [4] network of exponent $\gamma = -2.4$ and $N = 10^5$ nodes. We set the broadcast probability as $p = 10^{-4}$ and $T = 10^4$ so that each individual on average will have the minimal activity of one broadcast. The queue size has been extract from a Gaussian centered in q_{max}, that we set in the range between 50 and 300, and we set $\sigma = 10$. In the next sections we will describe the effect of each parameter in our model.

2.1 Effect of the Time Window T

One of the parameters of our model is the time window T during which we study the dynamics. This parameter regulates the maximum number of messages that will circulate in the network. In the first time steps the first messages will start to being sent among users and the queues start

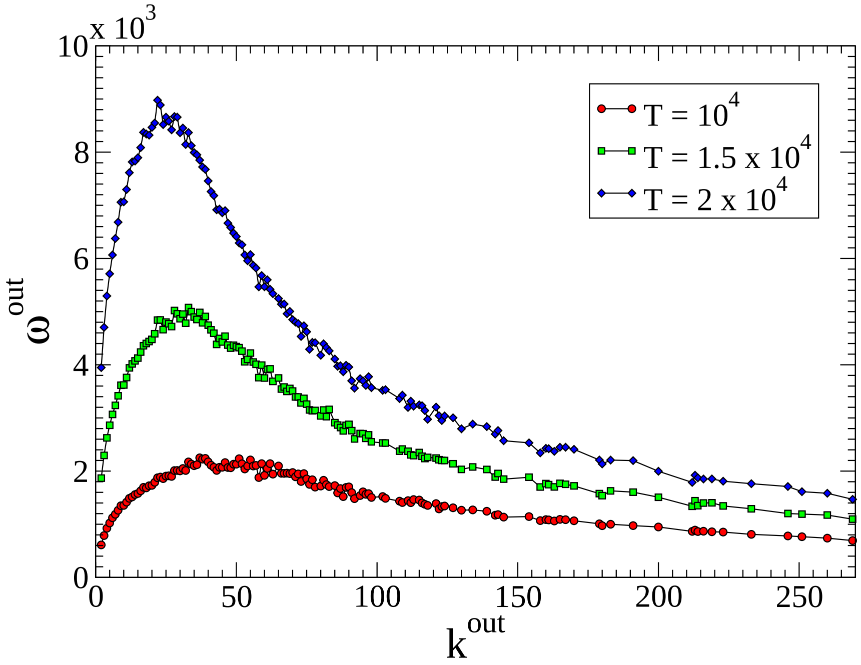

Fig. S 2: ω^{out} as a function of k^{out} for $q_{max} = 100$, $\sigma = 10$, scale-free network with $\gamma = -2.4$ and different values of T. We present the medians over 500 runs.

to get messages in and out. After a while we can aspect that the system reaches a dynamical equilibrium. In Fig. S (2) we show the behavior of our observable ω^{out} for different values of T, in particular we chose $T = 10^4, 1.5 \times 10^4, 2 \times 10^4$. The effect of time is clearly a shift on the y axis and a small change in the position of the peak. The first effect is due to the fact that the number of messages circulating in the systems increase linearly with T. The second effect is due to the reduction of fluctuations when more messages are sent. The peak becomes more clear and defined.

2.2 Effect of Broadcast Probability p

The effect of the broadcast probability is different on respect to the effect of the time window T. First of all our observable ω^{out} is linearly proportional to T in all regimes of k^{out} this is not true for p. The effect of p is crucial for users with a small number of contacts. As the p increases they will receive more messages and their activity will increase too, this does not occur in the other limit. When the saturation takes place the ω^{out} becomes completely independent of p. As show in details in a mean-field approach (Section (2.4)) for values of k^{out} small with respect to the queue size, ω^{out} scales linearly with p. Instead for a number of contacts much bigger than the queue size

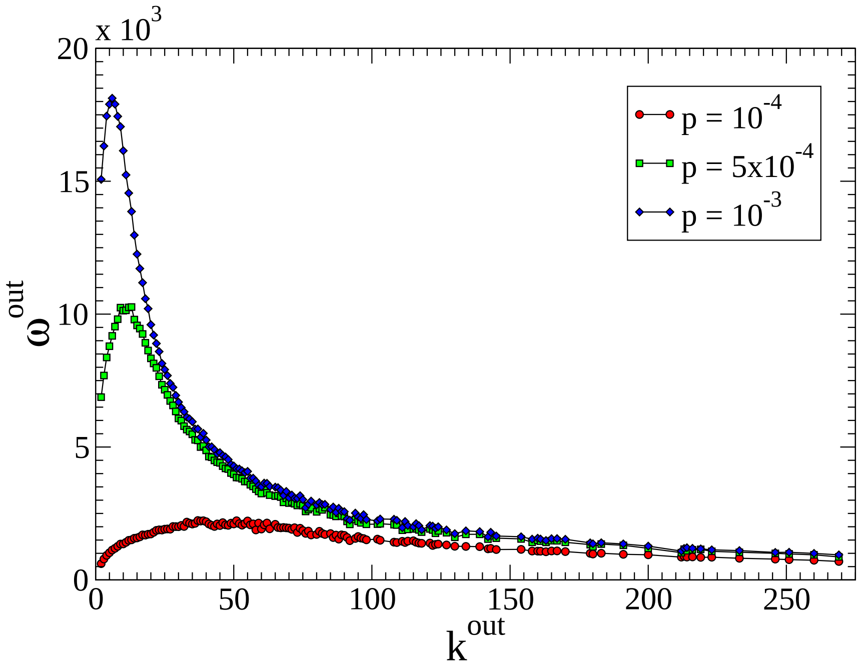

Fig. S 3: ω^{out} as a function of k^{out} for $q_{max} = 100$, $\sigma = 10$, scale-free network with $\gamma = -2.4$, $T = 10^4$ and different values of p. We present the medians over 500 runs.

ω^{out} is independent of p. These considerations are validated by our simulations as shown in Fig. S (3). We see a clear dependence on p for small values of k^{out} instead the same behavior for bigger values of k^{out}.

2.3 Effect of Network's Properties

Inspired by several studies [3, 5, 6, 2] we fix the baseline of our model using scale-free networks. It is important then to study how differences in the network structure affect the results. In this section we consider the effect of the exponent γ. As show in Fig. S (4) we run our model on top of scale-free networks with $\gamma = -2.2, -2.4, -2.6, -2.8$. As clear from the plot for smaller values of γ (bigger value in absolute value) gaps on k^{out} start to emerge. These are due to the network structure. The shape and position of the peak is the same for all the curves. The differeces are evident just on the peak height that increase as γ decreases. This is due the different redistribution of degrees and to the fact that with small γ the selection effect is more and more important. So we can say that the result are robust on γ.

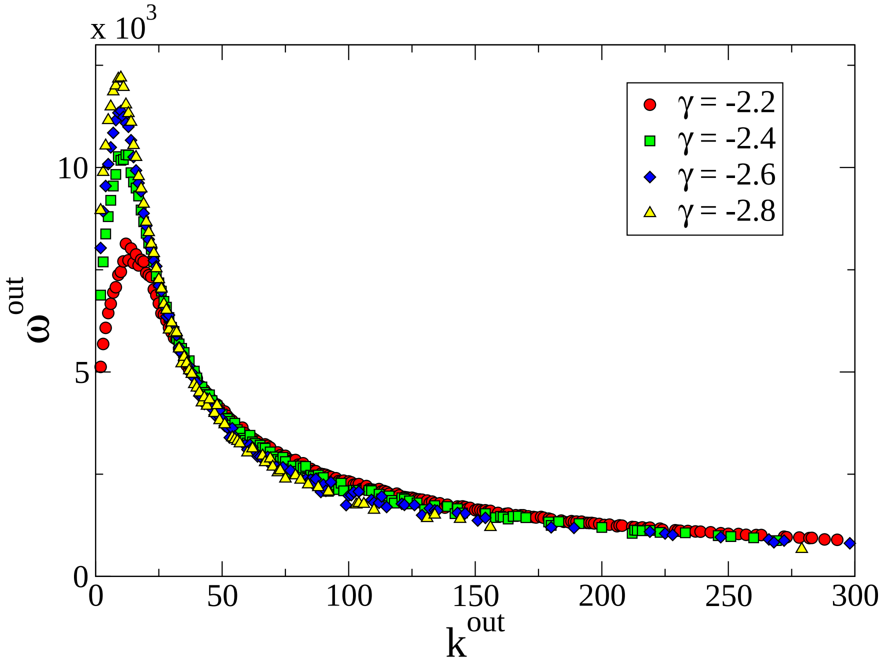

Fig. S 4: ω^{out} as a function of k^{out} for $q_{max} = 100$, $\sigma = 10$, $T = 10^4$, $p = 5 \times 10^{-4}$, scale-free network with different values of γ. We present the median over 500 runs.

2.4 Single User Analytics

As a way to better understand the mechanisms we describe, we analyzed the behavior of a single user i. In this mean-field approach its dynamics can be easily derived.
Let us consider k_i the degree of the user i and $q_{max,i}$ its queue size. It has a set of $k_i^{out} = k_i/2$ out-going links that it uses to send messages to its $k_i/2$ contacts and a set of $k_i^{in} = k_i/2$ in-coming through which it receives messages from it contacts. Its neighbors will have a priority k_j that we extract for a distribution $\mathcal{P}(k)$. The rules of our model are applied even in this single user case for T time steps. The probability that a neighbor j will send a message to the user i is:

$$p_{ji} = p + \frac{k_i}{< k > k_j}, \tag{3}$$

where as before p is the broadcast probability. The average number of messages that the user will receive at each time step t is then:

$$\langle R \rangle = \sum_j p_{ji} = pk_i^{in} + \frac{k_i}{< k >} \sum_{j \in k_i^{in}} \frac{1}{k_j}. \tag{4}$$

Since the number k_j are extracted from the same distribution the sum scales linearly with the number of element, k_i^{in}. For this reason we can write:

$$\langle R \rangle = \sum_j p_{ji} = p\frac{k_i}{2} + c\frac{k_i^2}{2 < k >}, \tag{5}$$

where c is a constant fixed by the distribution. The number of messages the user get from its contacts scale as the square of its degree, since the its priority is proportional to it as well as the number of in-coming connections.
Two different regimes are found: $k_i \ll q_{max,i}$ and vice versa.
In the first case the number of messages that the user will receive is small. It is not popular and in principle it can reply to all of them at each time step. We can consider that in this regime its queue is never completely full. Using the Equation (5) we get:

$$\omega_i^{out}(T) = \frac{1}{k_i^{out}} \mathcal{F}(R_1, R_2, \dots, R_T; q_{max,i}), \tag{6}$$

where $\mathcal{F}$ is a function of all the messages that are reicived by the users and its queue size, and R_t are the actual number of messages that the user reicive at the time t. It takes into account that fact that at each time step the number of sent replies is selected uniformly between 0 and the number of messages present in the queue. So after one time step the number of replies is:

$$S_1 = \xi_1 R_1, \tag{7}$$

where ξ_1 is a random number uniformly distribuited between 0 and 1. The number, S_2, of message that the user send at the second time step is a random fraction of the messages present in its queue:

$$S_2 = [R_1(1 - \xi_1) + R_2]\,\xi_2. \tag{8}$$

For $t = 3$ we get:

$$S_3 = \{[R_1(1-\xi_1) + R_2]\,(1-\xi_2) + R_3\}\,\xi_3 = [R_1(1-\xi_1)(1-\xi_2) + R_2(1-\xi_2) + R_3]\,\xi_3, \quad (9)$$

and so on. We can approximate these expressions using instead of the R_t the average value $\langle R \rangle$. For the general t it is easy to show how:

$$\begin{aligned} S_t &\sim \langle R \rangle \xi_t \left[1 + \sum_{j=1}^{t} \prod_{i=j}^{t-1} (1-\xi_i)\right] \\ &= \langle R \rangle \xi_t \left[2 - \xi_{t-1} + \mathcal{O}(\xi^2)\right] = \langle R \rangle \left[2\xi_t - \xi_t \xi_{t-1} + \mathcal{O}(\xi^3)\right]. \end{aligned} \quad (10)$$

The total number of messages sent is the numerator of ω^{out} and the sum of all the S_t:

$$\sum_{t=0}^{t=T} S_t \sim T \langle R \rangle, \quad (11)$$

Because each sum of product random numbers is order T. Using this we can write:

$$\omega_i^{out}(T) = \frac{\sum_{t=0}^{t=T} S_t}{k_i^{out}} \sim \frac{1}{k_i^{out}} T \left[p \frac{k_i^{out}}{2} + c \frac{(k_i^{out})^2}{2 < k >}\right] \sim T\ k_i^{out}. \quad (12)$$

In this regime we aspect then a linear increase with λ_i^{out} of the average number of replies per connections. As show in Fig. S (5) this is recover in the simulations.
The other regimes is found for a number of contacts bigger than the queue size. In this case the user at each time step gets a lot of messages and it is not able to handle all of it. The saturation process takes then place and it will reply to a small fraction of the total number of messages it is getting prioritizing them. At each time step this number is a random variable uniformly distributed between 0 and $q_{max,i}$. We can then write:

$$\omega_i^{out}(T) \sim \frac{1}{k_i^{out}} \sum_{t=0}^{T} \xi_t q_{max,i}. \quad (13)$$

The ξ_ts are random variable uniformly distributed between 0 and 1 so that each time step the number of replies is a random fraction of the queue size. For T large enough we get:

$$\omega_i^{out}(T) \sim \frac{T}{2k_i^{out}} q_{max,i}. \quad (14)$$

In this regime then we get a different scaling, typical of saturation problems. These arguments are in perfect agreement with the numerical results as shown in Fig. S (5).
We have shown two different regimes. A linear increasing behavior and a decreasing one. In the between of these opposite cases we will find a maximum of the function. The position of these peak is in general function of the queue size.

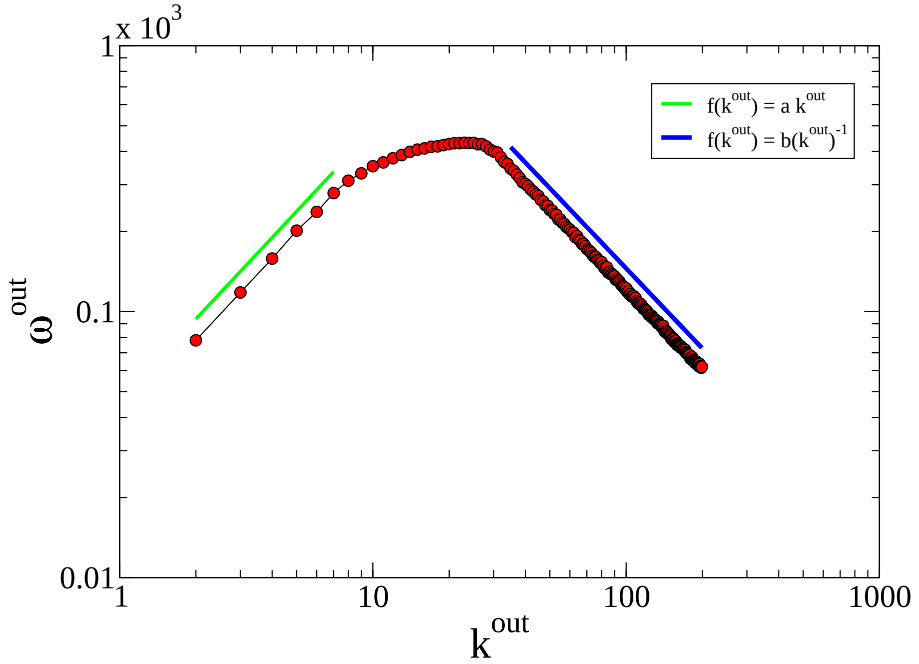

Fig. S 5: ω^{out} as a function of k^{out} for $q_{max,i} = 50$, $\sigma = 10$, $T = 10^3$ and $p = 10^{-3}$. Each point correspond to the median among 10^3 runs in which the dynamic of the single users has been implemented. The distribution of the priorities of the k_i neighbors are extracted from a power-law distribution with $\gamma = -2.1$.

References

[1] T. H. Cormen, C. E. Leiserson, R. L. Rivest, and C. Stein. *Introduction To Algorithms*. The MIT Press, 2nd edition, 2001.

[2] B. A. Huberman, D. M. Romero, and F. Wu. Social networks that matter: Twitter under the microscope. *First Monday*, 14:1, 2008.

[3] A. Java, X. Song, T. Finin, and B. Tseng. Why we twitter: understanding microblogging usage and communities. In *Proceedings of the 9th WebKDD and 1st SNA-KDD 2007 workshop on Web mining and social network analysis*, 2007.

[4] M. Molloy and B. Reed. A critical point for random graphs with a given degree sequence. *Random Structures and Algorithms*, 6:161, 1995.

[5] D. Boyd, S. Golder, and G. Lotan. Tweet, tweet, retweet: Conversational aspects of retweeting on twitter. In *43rd Hawaii International Conference on System Sciences*, page 412, 2008.

[6] C. Honeycutt and S. C. Herring. Beyond microblogging: Conversation and collaboration via twitter. In *Proceedings of the 42nd Hawaii International Conference on System Sciences*, 2008.
